# Supplementary material for: Effect of intrinsic foot muscles training on foot function and dynamic postural balance: A systematic review and meta-analysis
Source: PLoS One. 2022 Apr 20;17(4):e0266525. doi: 10.1371/journal.pone.0266525 (PMC9020712; doi:10.1371/journal.pone.0266525)
Supplement: S1 Table — (DOCX) [file pone.0266525.s001.docx]

**Searching strategy in Pubmed**

#1: (((((Foot muscle[Title/Abstract]) OR (Intrinsic foot muscle [Title/Abstract])) OR (Intrinsic flexor foot[Title/Abstract])) OR (Plantar muscle[Title/Abstract])) OR (Toe muscle[Title/Abstract])) OR (Hallux muscle[Title/Abstract])

#2: (((Training[Title/Abstract]) OR (Exercise[Title/Abstract])) OR (Strength[Title/Abstract])) OR (Strengthening[Title/Abstract])

#3: (((Foot function[Title/Abstract]) OR (Foot morphology[Title/Abstract])) OR (Foot structure[Title/Abstract])) OR (Foot posture[Title/Abstract])

#4: (((((((((Dynamic postural balance[Title/Abstract]) OR (Dynamic balance[Title/Abstract])) OR (Posture stability[Title/Abstract])) OR (Posture control[Title/Abstract])) OR (balance performance[Title/Abstract])) OR (balance test[Title/Abstract])) OR (balance function[Title/Abstract])) OR (balance ability[Title/Abstract])) OR (Postural[Title/Abstract])) OR (Balance[Title/Abstract])

#5: #1 AND #2 AND #3 AND #4
