# Supplementary material for: Effect of intrinsic foot muscles training on foot function and dynamic postural balance: A systematic review and meta-analysis
Source: PLoS One. 2022 Apr 20;17(4):e0266525. doi: 10.1371/journal.pone.0266525 (PMC9020712; doi:10.1371/journal.pone.0266525)
Supplement: S3 Table — (PDF) [file pone.0266525.s003.pdf]

To enable PROSPERO to focus on COVID-19 registrations during the 2020 pandemic, this registration record was automatically published exactly as submitted. The PROSPERO team has not checked eligibility.

Citation

Zhen Wei, Lin Wang. Effective of intrinsic foot muscle training on foot biomechanics and running-related injuries: A systematic review. PROSPERO 2021 CRD42021232984 Available from: https://www.crd.york.ac.uk/prospero/display_record.php?ID=CRD42021232984

Review question

The aim of this review is to holistically evaluate the effect of intrinsic foot muscle training on foot biomechanics and running-related injuries

Searches

A comprehensive search strategy will be used by the first author to search the following databases: PubMed, CINAHL, SPORT Discus and Web of Science. Additionally, manual searches of reference lists of eligible studies will be conducted. Studies will be limited to English languages without publication period restriction.

Types of study to be included

-Randomized control trials and pre/post-intervention studies will be included in this review

-Research specific to intrinsic foot muscles training as an intervention method

-Should have at least one desired foot biomechanics outcome or running injuries

Condition or domain being studied

Through specialized intrinsic foot muscles training, the improved foot strength was believed to help runners to prevent common running injuries. Numerous studies have explored the effect of intrinsic foot muscle training on foot biomechanics and running-related injuries, but there is still no consensus in the literature regarding the effective of this training method.

Therefore, the aim of this review is to critically evaluate the literature investigating intrinsic foot muscles training interventions.

Participants/population

Participants (both male and female) aged 18 years or older, without lower limb deformity.

Intervention(s), exposure(s)

Studies utilizing a specific plantar or intrinsic foot muscle training as intervention method will be included in this review.

Comparator(s)/control

Intrinsic foot muscle training (i.e. short foot exercise) was included as one group when comparing with other forms of intervention or group received nothing.

Main outcome(s)

Foot biomechanics can include but not limited to the following: navicular height; arch height index; navicular drop; truncated foot length; foot muscle strength.

Running-related Injuries: any musculoskeletal pain or injury caused by running practice that induces changes

in the form, duration, intensity, or frequency of training for at least 1 week.

Measures of effect

We used Cohen's d effect sizes (ES) with associated 95% confidence interval (CI) to perform statistical analysis.

Additional outcome(s)

None

Measures of effect

None

Data extraction (selection and coding)

Full text of potentially eligible articles will be independently screened by two authors. Any disagreement will be resolved by a third author.

The following data will be extracted:

1) study background (authors, year, study design)

2) population characteristics (demographics, male/female ratio)

3) interventions characteristics i.e. exercise prescription (sets / repetitions)

4) outcome characteristics

Where information is unclear, the corresponding author of the study will be contacted via email for clarification.

Risk of bias (quality) assessment

Two examiners will assess the risk of bias for all articles using Physiotherapy Evidence Database scale (PEDro Scale) independently. Disagreements were resolved by third party adjudication.

Strategy for data synthesis

Due to the heterogeneous nature of the anticipated studies for review, we will pool results of studies using the generic inverse variance method in Review Manager (RevMan 5.3). We calculated the outcomes with 95% confidence intervals using the fixed-effect model. Where there was substantial statistical heterogeneity we pooled the data using the random-effects model.

Analysis of subgroups or subsets

N/A

Contact details for further information

Zhen Wei
weizhen443@163.com

Organisational affiliation of the review

School of Kinesiology, Shanghai University of Sport
http://yk.sus.edu.cn/

Review team members and their organisational affiliations

Mr Zhen Wei. School of Kinesiology, Shanghai University of Sport
Mr Lin Wang. Shanghai university of sport

Type and method of review

Systematic review

Anticipated or actual start date

05 February 2021

Anticipated completion date
01 February 2022

Funding sources/sponsors
The National Natural Science Fund of China

Conflicts of interest

Language
English

Country
China

Stage of review
Review Ongoing

Subject index terms status
Subject indexing assigned by CRD

Subject index terms
MeSH headings have not been applied to this record

Date of registration in PROSPERO
08 March 2021

Date of first submission
05 February 2021

Stage of review at time of this submission
The review has not started

Stage	Started	Completed
Preliminary searches	No	No
Piloting of the study selection process	No	No
Formal screening of search results against eligibility criteria	No	No
Data extraction	No	No
Risk of bias (quality) assessment	No	No
Data analysis	No	No

The record owner confirms that the information they have supplied for this submission is accurate and complete and they understand that deliberate provision of inaccurate information or omission of data may be construed as scientific misconduct.
The record owner confirms that they will update the status of the review when it is completed and will add publication details in due course.

Versions
08 March 2021

08 March 2021
