# Supplementary material for: Effect of intrinsic foot muscles training on foot function and dynamic postural balance: A systematic review and meta-analysis
Source: PLoS One. 2022 Apr 20;17(4):e0266525. doi: 10.1371/journal.pone.0266525 (PMC9020712; doi:10.1371/journal.pone.0266525)
Supplement: Attachment — Submitted filename: Response to Reviewers.docx [file pone.0266525.s004.docx]

**Dear Editor**

On behalf of our research team, we would like to thank the editor for giving us a chance to resubmit the manuscript (PONE-D-21-23366), and also thank the reviewers for giving us constructive suggestions, which would help us both in English and in depth to improve the quality of our study. Here we submit a new version of our manuscript with the title “Revised Manuscript with Track Changes”, which has been modified according to the reviewers’ suggestions. Efforts were also made to correct the mistakes and improve the English of the manuscript. Revisions were highlighted using **red font** in manuscript. We also included our responses to the various points raised by the reviewer below.

We are looking forward to your reply.

Thank you.

Yours sincerely,

Lin Wang, MB, PhD

Professor,

Department of Sports Rehabilitation, Shanghai University of Sport

Shanghai, China.

Tel: (86) 21 5125 3426 Fax: (86) 21 5125 3470

Email: wanglin@sus.edu.cn

**Reviewer reports:**

## Journal requirements:

When submitting your revision, we need you to address these additional requirements.

1. Please ensure that your manuscript meets PLOS ONE's style requirements, including those for file naming. The PLOS ONE style templates can be found at

<https://journals.plos.org/plosone/s/file?id=wjVg/PLOSOne_formatting_sample_main_body.pdf> and

<https://journals.plos.org/plosone/s/file?id=ba62/PLOSOne_formatting_sample_title_authors_affiliations.pdf>

A: Thank you for your suggestion. We have modified our manuscript in according with PLOS ONE's style requirements.

2. We note that the grant information you provided in the ‘Funding Information’ and ‘Financial Disclosure’ sections do not match.

When you resubmit, please ensure that you provide the correct grant numbers for the awards you received for your study in the ‘Funding Information’ section.

A: Thank you for your suggestion. We have corrected it.

Funding Information: This work was supported by the National Natural Science Foundation of China [11572202]

3. Thank you for stating the following financial disclosure:

 “This work was supported by the National Natural Science Foundation of China [11572202]”

Please state what role the funders took in the study.  If the funders had no role, please state: "The funders had no role in study design, data collection and analysis, decision to publish, or preparation of the manuscript."

If this statement is not correct you must amend it as needed.

Please include this amended Role of Funder statement in your cover letter; we will change the online submission form on your behalf.

A: Thank you for your suggestion. The funder supported this study during data collection and analysis. We have added it in the cover letter.

4. Thank you for stating the following in the Funding Section of your manuscript:

“This work was supported by the National Natural Science Foundation of China [11572202”

We note that you have provided information within the Funding Section. Please note that funding information should not appear in other areas of your manuscript. We will only publish funding information present in the Funding Statement section of the online submission form.

Please remove any funding-related text from the manuscript and let us know how you would like to update your Funding Statement. Currently, your Funding Statement reads as follows:

“This work was supported by the National Natural Science Foundation of China [11572202]”

Please include your amended statements within your cover letter; we will change the online submission form on your behalf.

A: Thank you for your suggestion. We have removed the funding information in the manuscript and added it in cover letter.

5. In your Data Availability statement, you have not specified where the minimal data set underlying the results described in your manuscript can be found. PLOS defines a study's minimal data set as the underlying data used to reach the conclusions drawn in the manuscript and any additional data required to replicate the reported study findings in their entirety. All PLOS journals require that the minimal data set be made fully available. For more information about our data policy, please see http://journals.plos.org/plosone/s/data-availability.

Upon re-submitting your revised manuscript, please upload your study’s minimal underlying data set as either Supporting Information files or to a stable, public repository and include the relevant URLs, DOIs, or accession numbers within your revised cover letter. For a list of acceptable repositories, please see http://journals.plos.org/plosone/s/data-availability#loc-recommended-repositories. Any potentially identifying patient information must be fully anonymized.

Important: If there are ethical or legal restrictions to sharing your data publicly, please explain these restrictions in detail. Please see our guidelines for more information on what we consider unacceptable restrictions to publicly sharing data: http://journals.plos.org/plosone/s/data-availability#loc-unacceptable-data-access-restrictions. Note that it is not acceptable for the authors to be the sole named individuals responsible for ensuring data access.

We will update your Data Availability statement to reflect the information you provide in your cover letter

A: Thank you for your suggestion. All data are fully available without restriction.

6. PLOS requires an ORCID iD for the corresponding author in Editorial Manager on papers submitted after December 6th, 2016. Please ensure that you have an ORCID iD and that it is validated in Editorial Manager. To do this, go to ‘Update my Information’ (in the upper left-hand corner of the main menu), and click on the Fetch/Validate link next to the ORCID field. This will take you to the ORCID site and allow you to create a new iD or authenticate a pre-existing iD in Editorial Manager. Please see the following video for instructions on linking an ORCID iD to your Editorial Manager account: https://www.youtube.com/watch?v=_xcclfuvtxQ

A: Thank you for your suggestion. We have updated corresponding author’s Information.

Also, the ORCID ID of first and corresponding author are as follows:

Zhen Wei: https://orcid.org/0000-0002-5938-5019

Lin Wang: https://orcid.org/0000-0003-3057-3742

## 5. Review Comments to the Author

### Reviewer #1:

The authors present a systematic review study on the effectiveness of interventions that aim to improve the functioning of the Intrinsic Foot Muscles. This work has potential relevance in the prevention of foot and ankle injuries and provides a better understanding of the functional anatomy of the foot and the effects of training thereon. The manuscript overall is of sound quality, however it needs some strict improvements in the methods section in order to become eligible for publication.

A: Thank you for your positive comments. Your comments were highly insightful and help us improve the quality of the manuscript. We have modified the methods section.

MAJOR ISSUES

Aims. The aim of the current study is somewhat unclear: “Nonetheless, current studies on IFM training are cross-sectional, and evidence to support this claim is lacking. Therefore, the aim of this systematic review is to identify and determine the effect of IFM training on foot function and dynamic postural balance” (line 48-50).

This needs to be further clarified. If all past studies are cross sectional, what will be the added value of this review? In fact, later you find 13 RCTs that have been done. Why do these not feature in the introduction?

A: Thank you for your suggestion. This sentence needs further consideration and we have rewritten it as follows:

“On the basis of previous findings, IFMs training can ameliorate foot biomechanic variables, resulting in the minimization of an accumulation of repetitive stresses and higher absorption of ground reaction force. Although numerous studies explore the effect of IFMs training, there is still lacking of evidence-based studies to review it systematically. Therefore, this systemic review with meta-analysis aimed to identify and determine the IFMs training on foot function and dynamic postural balance.” (Page 3 Line 51-55)

This issue is further confused at the start of the discussion where the aim seems to have been to summarize the current studies. Furthermore, judging from the methods and the results, the aim seems to have been to assess the effect of IFM training on foot biomechanical outcomes using a meta-analysis. Please clarify.

A: Thank you for your suggestion. We have modified it as follows:

“This systematic review summarized the current studies that explored the effect of IFMs training on foot biomechanical outcomes using a meta-analysis.” (Page 16 Line 229-230)

Methods. The major issues of the current manuscript lie in the methods section. Although the authors provide an adequate description of the methods to see that their approach is well thought out, there is insufficient details, and sometimes inconsistencies, that limit the reproducibility of the approach. Relating to this, the following issues will need to be addressed:

1. In the description of the search (line 57-60), it would be good to add some specification of the BOOLEAN operators used in the search.

A: We have added specification of the BOOLEAN operators in the manuscript. (Page 3 Line 62-67)

2. In the supplementary file specifying the search, it should be specified what type of search was performed (e.g. title/abstract/keywords, topic, mesh terms) and it should be specified how the search was adapted to the different search engines.

A: Thank you for your suggestion. We have revised it and given an example of search strategy for PubMed database.

3. There is some confusion in the use of quotation marks. In the main text, single quotation marks are used, in the supplementary file these are double marks. Please specify what was used in the searches, as these have a different meaning in the search engines.

A: Thank you for your suggestion. We have corrected the quotation marks. (Page 3 Line 62-67)

4. The authors note the inclusion criterium ‘Research specific to IFM training as an intervention’ (line 66). As the authors mention, these muscles have a function many whole-body activities (e.g. walking), so arguably can be trained with many whole body activities as well (e.g. walking, running, whole body vibration training). How was IFM training defined for inclusion in the review?

A: Thank you for your suggestion. IFMs training was defined as “training program emphasizing the neuromuscular recruitment of the plantar intrinsic foot muscles, like short foot exercise.” We have added it in the manuscript. (Page 4 Line 71-73)

5. No limitations were introduced to the search dates and yet only articles from after 2013 were included. This might just result from the novelty within the research field, but it could also indicate a fatal flaw in the search strategy. That is, it might be that the field evolved and that now different terms are used compared to 20 years ago. On example might be the following study: DOI: 10.1123/jsr.21.4.327. Please comment on this issue.

A: Thank you for your suggestion. In order to keep the novelty within the research field, we only included studies from 1 January 2011 to 14 February 2021. We have updated the search dates. (Page 3 Line 60-61)

For DOI: 10.1123/jsr.21.4.327, the main aim of this study was comparing the short-foot exercise and towel-curl exercise, rather than explore the effect of IFMs training on foot function or dynamic postural balance, so we excluded it.

6. The authors state ‘When possible, the results of the included studies were pooled for meta-analysis using RevMan 5.3.’ (line 84). How was it determined whether this was possible? It would be helpful to inform the reader on this process. How come only 6 of the 15 papers ended up in the meta-analysis?

A: Thank you for your suggestion. Because of the foot biomechanics are various, after reviewing all included articles, only some common parameters can be pooled.

We have modified the sentence to “The pooled mean difference of training effects was calculated and illustrated by forest plots with Review Manager version 5.3. Random effects models were used to calculate standardized mean differences and 95% confidence intervals (CIs) for the control and experimental groups.” (Page 5 Line 92-94)

7. The prospero registration introduces the usage of the PEDro scale, in the methods section a ‘Checklist for Measuring Quality’ is introduced and in the results the Modified Downs and Black Quality Index is reported. Please resolve these inconsistencies and please specify why the prospero protocol was not adhered to.

A: Thank you for your suggestion. ‘Checklist for Measuring Quality’ is refers to the ‘Modified Downs and Black Quality Index’. We have corrected it. Additionally, the study was registered in PROSPERO (CRD42021232984), we added it in the supporting information. (Page 4 Line 84)

MINOR ISSUES

1.The use of abbreviations drastically limits the readability of the manuscript. Following PLOS one author guidelines, the use of abbreviations should be limited, and non-standard abbreviations should not be used unless they appear at least three times in the text. While I believe that most abbreviations are used more than thrice, this is mainly through their use in table 2. I would advise to limit abbreviating terms in the main text and only use abbreviations in the table.

A: Thank you for your suggestion. We have modified the abbreviated terms in the main text.

2. Line 22. I would suggest to use the term ‘effector’ rather than ‘organ’

A: We have revised " organ " as " effector ".

3. Line 27. With the introduction of IFMs, I would advise to name which muscles are meant specifically.

A: We have named the potential muscles specifically. (Page 2 Line 29-30)

4. Line 39-40. With introduction of the training methods, it would be helpful if the authors could explain in a bit more detail what these training methods are about and in particular where they are different from each other.

A: Thank you for your suggestion. We have added details about the training methods as follows:

“Several methods can be used to train IFMs, such as short foot exercise (SFE), toe-posture exercises, towel curl exercises and metatarsophalangeal joint (MPJ) muscle training [17-21], among which, SFE was studied most because of it characteristics utilizing the IFMs to draw the metatarsal heads back towards the heel whilst minimizing distal interphalangeal flexion” (Page 2 Line 43-46)

5. Line 65-66. “The remaining studies were then reviewed as full texts on the basis of the following criteria: …” - The present wording makes it appear like the title and abstract screening were based on different criteria. Please clarify whether this was the case.

A: Thank you for your suggestion. Title, abstract and full texts screening all based on the same criteria.

We have revised the sentence to “After removing duplicate articles, the search results were screened independently by two authors according to titles, abstracts and full texts on the basis of the following criteria.” (Page 3 Line 70-71)

6. Line 91-92. “After full text screening, one article as clinical pearl was excluded” – personally I am unfamiliar with the term ‘clinical pearl’ and why this should lead to exclusion. This might be just me, but in any case a bit further clarification might be helpful.

A: Thank you for your suggestion. The relative references have been added in the text.

*REF: Vincent KR, Vincent HK. Use of Foot Doming for Increasing Dynamic Stability and Injury Prevention in Runners and Athletes. Curr Sports Med Rep, 2018;17(10):320-321.*

7. Line 135. Descriptive stats are only provided for runners. How about the non-runner participants?

A: Thank you for your suggestion. After re-reviewed the included studies, we added descriptive data of participants as follows:

“The 15 studies included a total of 610 participants. Six studies included elite long-distance runners (n=328) [17, 18, 22, 23, 37, 38]. Three studies explored IFMs on pes planus patients (n=75) [11, 35, 39], and six other studies only included healthy or asymptomatic subjects (n=207) [10, 16, 19, 25, 27, 36].” (Page 8 Line 152-154)

8. Line 148-149. The information about intervention duration is duplicated after section 3.2 and does not need to be mentioned here.

A: We have removed it.

9. Line 153. Please define extrinsic foot muscles.

A: Thank you for your suggestion. The extrinsic foot muscles are around the lower leg and act to plantarflexion dorsiflexion, invert and evert the foot. We have modified it. (Page 9 Line 171)

10. Line 169. Please define CSA

A: We have revised CSA as cross-sectional area

11. Line 199. Please provide a more extensive/descriptive caption for figure 4.

A: We have provided descriptive caption for figure 4.

Figure 4. Funnel plots showing publication bias among studies used to compare IFMs training and control groups (Page 11 Line 226-227)

12. Line 216. The authors mention advance technologies. Can they provide some example of such technologies?

A: We have provided example of advance technologies as follows:

“future studies should utilise more advanced technology, like magnetic resonance imaging to measure the IFMs fat infiltration and cross-sectional area after training.” (Page 17 Line 242-243)

13. Line 238. I believe this should be ‘though’ instead of ‘through’.

A: We have revised " through’" as " though".

### Reviewer #2:

The goal of this manuscript was to develop a systematic review and a meta-analysis systematic review to identify the effect of intrinsic foot muscles training on foot function and dynamic postural balance.

The manuscript is relatively well grounded, it deals with a relevant topic to be investigated and deserves to be published. In its current state, the level of English throughout your manuscript need careful revision. I suggest a fluent English speaker read and correct grammatical, word choice, and syntax errors throughout the manuscript.

Although paper is straightforward but there are many suggestions/comments that I addressed throughout the manuscript.

A: Thank you for your positive comments. Your comments were highly insightful and help us improve the quality of the manuscript. The level of English throughout our manuscript have been carefully revised. A native speaker was invited to edit the manuscript.

There is a critical point in the methodology that is pooling data from studies that used different intervention approaches and time of intervention, and thus, the results are not expected to be the same. Pooling the data into a meta-analysis, it groups studies even if the results are not coming from the same approach of IFM training. Pooling data that came from different interventions, exercises and times of intervention and populations lead to huge heterogeneity that will certainly compromise a meta-analysis. It is pivotal to choose same interventions to be included in a meta-analysis and only then we could have an unbiased statistical analysis. This should be acknowledged and maybe exclude the meta-analysis from the manuscript. it does not add much as the only two variables that could be pooled did not achieve power enough to conclude anything robust.

A: Thank you for your suggestion. Given that pooling the data that came from different methods may lead to huge heterogeneity and compromise a meta-analysis, after review the included studies again, only two variables that could be pooled in the current studies.

Language issues examples (there are other weird sentences and word choices that need revision):

1. Please, consider reviewing the English language and grammar by a native reviewer. The writing compromises the understanding of parts of the manuscript.

A: Thank you for your suggestion. The manuscript language and grammar have been carefully revised by a native reviewer.

2. Introduction: “Whilst running, the feet act as the starting body parts…” – correct to body PART.

A: We have corrected it. (Page 2 Line 24)

3. Introduction: “Given that the feet are the most proximal aspect of the lower limb…”- correct to most DISTAL. The feet are distal and not proximal part of LL.

A: Thank you for your suggestion. We have corrected it. (Page 2 Line 27)

4. Introduction: “ground impact force (GRF)…” – correct to ground REACTION force.

A: We have corrected it. (Page 2 Line 28)

5. Introduction: “During the early stance phase in running…” – correct to OF REARFOOT STRIKE running

A: We have corrected it. (Page 2 Line 36)

6. Introduction: “… and providing better propulsion for runners in the swing phase [12].” – it provides better propulsion in the PUSH OFF phase, not swing phase!

A: Thank you for your suggestion. This is a typo. We have corrected it. (Page 2 Line 40)

Abstract and Introduction

1. Please, provide the PROSPERO registry number in the abstract.

A: Thank you for your suggestion. We have added registry number in the abstract. (Page 1 Line 15)

2. At the end of the second paragraph of the introduction, the authors provide a closing sentence concluding all previous sentences, however it does not make sense. “Therefore, strengthening of IFMs has been suggested for the prevention and treatment of foot and ankle sports injuries [16].” The previous sentences describe all important roles of the foot and its intrinsic muscles, but they do not provide any evidence that strengthening the intrinsic foot muscles would prevent injuries. It is a fallacy and must be amended.

A: Thank you for your suggestion. We have amended it.

3. Line 39: Please consider including other papers that presented different foot muscle exercises program, as Matias et al. BMC Musculoskelet Disord. 2016 doi: 10.1186/s12891-016-1016-9. and Ferreira et al. Trials. 2020 doi: 10.1186/s13063-019-4017-9.

A: Thank you for your suggestion. We have added the relative references in the text.

4. Lines 43-44: “These factors are believed to protect runners from common running-related injuries [21, 23]” it is an incorrect affirmation. Reference 21 does not “believe”, it proved that foot-related exercises do prevent RRI and reference 23 did not study RRI. Consider rephrasing the sentence.

A: Thank you for your suggestion. We have rephrased this sentence as follows:

“which proved that foot-related exercises do prevent running-related injuries [23]” (Page 3 Line 48)

5. Lines 47-48: “… and transmission efficiency of GRF.” What did the authors mean by efficiency GRF?! It does not make sense the way it is written. Please, rephrase the sentence.

A: Thank you for your suggestion. We have rephrased this sentence as follows:

“resulting in the minimization of an accumulation of repetitive stresses and higher absorption of ground reaction force.” (Page 3 Line 52-53)

6. Lines 48-49: “Nonetheless, current studies on IFM training are cross-sectional, and evidence to support this claim is lacking.” This sentence is also a fallacy. There are many papers that as RCT and not cross-sectional and those RCTs have proven the efficacy of the foot muscles training for several conditions Just to name two: Mølgaard CM, J Sci Med Sport. 2018 doi: 10.1016/j.jsams.2017.05.019. And your references 10 and 21, among other that need to be reviewed.

A: Thank you for your suggestion. This sentence needs further consideration and we have rewritten it as follows:

“On the basis of previous findings, IFMs training can ameliorate foot biomechanic variables, resulting in the minimization of an accumulation of repetitive stresses and higher absorption of ground reaction force. Although numerous studies explore the effect of IFMs training, there is still lacking of evidence-based studies to review it systematically. Therefore, this systemic review with meta-analysis aimed to identify and determine the IFMs training on foot function and dynamic postural balance.” (Page 3 Line 52-55)

7. It is not clear what is the aim of this systematic review: look for RCTs, since the authors criticized that the majority of current studies are cross-sectional, or focuses on all types of studies. This need to be clear in the introduction.

A: Thank you for your suggestion. Some sentence needs further consideration in the introduction and we have rephrased this part. (Page 3 Line 52-55)

8. The main question investigated in the review must be clearly posted in the introduction and it is not clear at all.

A: Thank you for your suggestion. We have rewritten the review question clearly.

Methods

1.The type of studies that were searched and included need to be described in the search strategy.

A: Thank you for your suggestion. The type of studies has been added in the search strategy. (Page 3 Line 66)

2. What is a “desired foot biomechanics outcome” itemized in (2) in the Study selection session.

A: Thank you for your suggestion. “biomechanics outcome” means common biomechanics parameters in the studies. We have corrected it. (Page 4 Line 73)

3. Please, specify better what “outcome characteristics” are in the data extraction session.

A: Thank you for your suggestion. In the “outcome characteristics”, we have modified it. (Page 4 Line 79)

4. There is a critical point in the methodology that is pooling data from studies that used different intervention approaches and time of intervention, and thus, the results are not expected to be the same. Pooling the data into a meta-analysis, it groups studies even if the results are not coming from the same approach of IFM training. This should be acknowledged and maybe exclude the meta-analysis of the manuscript. Or, at least, include as a limitation of the meta-analysis performed.

A: Thank you for your suggestion. We admitted that different intervention approaches and time of intervention may contribute to the results different from what we expected. We have modified it in the limitation. (Page 19 Line 281-282)

5. Figure 1 need to clearly describe the reasons for excluding the 204 papers from the review.

A: We have described the reasons for excluding papers clearly. (Page 6 Line 116-119)

Results

1.“one article as clinical pearl…” What the authors mean by that?

A: Thank you for your suggestion. The relative references have been added in the text.

*REF: Vincent KR, Vincent HK. Use of Foot Doming for Increasing Dynamic Stability and Injury Prevention in Runners and Athletes. Curr Sports Med Rep, 2018;17(10):320-321.* (Page 5 Line 100-101)

2.When describing the meta-analysis, the muscle volume that changed in one study did not appear and should at least be mentioned, not only the CSA, but muscle volume. The way it looks, readers will not know IFM changes foot muscles volume.

A: Thank you for your suggestion. We have added muscle volume change in the manuscript. (Page 9 Line 187)

3. Please, correct Weight to body mass, because you express in kg.

A: We have corrected descriptions Weight to body mass.

4. Table 3 – include descriptions of abbreviations in the caption: FPI, COM, INT and BMI.

A: Thank you for your suggestion. We have included descriptions of abbreviations.

Discussion

1.Overall, the discussion is straightforward and focused, but the English language that needs revision.

A: Thank you for your suggestion. We have asked for native English speaker to revise our manuscript.

2.Lines 208-216: it is important to emphasizes that the studies did see differences after the intervention in CSA and volume of the foot muscles. But the heterogeneous and critical meta-analysis performed did not detect any difference probably due to pooling papers with completely different interventions, exercises and times of intervention and populations.

A: Thank you for your suggestion. Some studies may be see differences after the intervention in CSA and volume of the foot muscles. But the heterogeneous and critical meta-analysis performed actually did not detect any difference.

3. The same comment above can be applied to third, fourth, fifth paragraphs of the discussion.

A: Thank you for your suggestion. IFMs training may bring some positive effect of foot biomechanics parameters, but after the pooled data analysis of the included studies, the actual effect of strength training on IFMs was various.
