# Supplementary material for: Effect of intrinsic foot muscles training on foot function and dynamic postural balance: A systematic review and meta-analysis
Source: PLoS One. 2022 Apr 20;17(4):e0266525. doi: 10.1371/journal.pone.0266525 (PMC9020712; doi:10.1371/journal.pone.0266525)
Supplement: Attachment — Submitted filename: Response to Reviewers.docx [file pone.0266525.s005.docx]

**Dear Editor**

On behalf of our research team, we would like to thank the editor for giving us a chance to resubmit the manuscript (PONE-D-21-23366), and also thank the reviewers for giving us constructive suggestions, which would help us both in English and in depth to improve the quality of our study. Here we submit a new version of our manuscript with the title “Revised Manuscript with Track Changes”, which has been modified according to the reviewers’ suggestions. Efforts were also made to correct the mistakes and improve the English of the manuscript. Revisions were highlighted using **red font** in manuscript. We also included our responses to the various points raised by the reviewer below.

We are looking forward to your reply.

Thank you.

Yours sincerely,

Lin Wang, MB, PhD

Professor,

Department of Sports Rehabilitation, Shanghai University of Sport

Shanghai, China.

Tel: (86) 21 5125 3426 Fax: (86) 21 5125 3470

Email: wanglin@sus.edu.cn

## Comments to the Author

1. If the authors have adequately addressed your comments raised in a previous round of review and you feel that this manuscript is now acceptable for publication, you may indicate that here to bypass the “Comments to the Author” section, enter your conflict of interest statement in the “Confidential to Editor” section, and submit your "Accept" recommendation.

Reviewer #2: (No Response)

2. Is the manuscript technically sound, and do the data support the conclusions?

The manuscript must describe a technically sound piece of scientific research with data that supports the conclusions. Experiments must have been conducted rigorously, with appropriate controls, replication, and sample sizes. The conclusions must be drawn appropriately based on the data presented.

Reviewer #2: Yes

3. Has the statistical analysis been performed appropriately and rigorously?

Reviewer #2: Yes

4. Have the authors made all data underlying the findings in their manuscript fully available?

The PLOS Data policy requires authors to make all data underlying the findings described in their manuscript fully available without restriction, with rare exception (please refer to the Data Availability Statement in the manuscript PDF file). The data should be provided as part of the manuscript or its supporting information, or deposited to a public repository. For example, in addition to summary statistics, the data points behind means, medians and variance measures should be available. If there are restrictions on publicly sharing data—e.g. participant privacy or use of data from a third party—those must be specified.

Reviewer #2: Yes

5. Is the manuscript presented in an intelligible fashion and written in standard English?

PLOS ONE does not copyedit accepted manuscripts, so the language in submitted articles must be clear, correct, and unambiguous. Any typographical or grammatical errors should be corrected at revision, so please note any specific errors here.

Reviewer #2: No

6. Review Comments to the Author

Please use the space provided to explain your answers to the questions above. You may also include additional comments for the author, including concerns about dual publication, research ethics, or publication ethics. (Please upload your review as an attachment if it exceeds 20,000 characters).

A: Thank you for your efficient work in reviewing our manuscript. Your comments were highly insightful and help us improve the quality of the manuscript. We have improved the English of the manuscript, and would like to re-submit it for your consideration.

Reviewer #2:

1. Although most of the previous comments from this reviewer were addressed properly, the paper still needs improvement in language and flow. It is impossible to follow some new additions. I strongly recommend a professional service for proofreading, not only a colleague who is a native speaker, because it clearly did not work this time. The way the paper reads it is impossible to be fully understood.

A: Thank you for your suggestion. We have modified the English language of the manuscript.

2. I have used the search strategies now described in the paper and found some very important quality papers (PEDro scale 5 to 8) that were not included in this review. Additionally, I am familiar with many papers that used IFM training and it matched some of them that I found but is it no in this manuscript. I am listing below for the authors to consider including as it must not be out of your systematic review.

Missing relevant papers regarding you research question, your outcomes and your chosen study designs:

I. (PEDro score 7) Lee D-R, Choi Y-E. Effects of a 6-week intrinsic foot muscle exercise program on the functions of intrinsic foot muscle and dynamic balance in patients with chronic ankle instability. Journal of exercise rehabilitation. 2019;15(5):709.

Specifically, you cite this study but I did not understand why it was not included in your systematic review.

A: Thank you for your suggestion. Given to the fact that patients with chronic ankle instability may have different foot biomechanics, and IFMs training has less effect on the ankle joint. So after negotiation between the two authors, we finally decided excluded this article.

II. (PEDro score 5) Day EM, Hahn ME. Increased toe-flexor muscle strength does not alter metatarsophalangeal and ankle joint mechanics or running economy. Journal of sports sciences. 2019;37(23):2702-10.

A: Thank you for your suggestion. After reexamined all articles, we found this article meets our inclusion criteria. We have added it and rewrite our manuscript.

III. (PEDro score 8) Jung D-Y, Koh E-K, Kwon O-Y. Effect of foot orthoses and short-foot exercise on the cross-sectional area of the abductor hallucis muscle in subjects with pes planus: a randomized controlled trial. Journal of back and musculoskeletal rehabilitation. 2011;24(4):225-31.

A: Thank you for your suggestion. Because of this article mainly compare foot orthosis (FO) group and the group combined foot orthosis and short-foot exercise (FOSF) after 8-week intervention. We cannot rule out the effect of foot orthosis on the cross-sectional area of the abductor hallucis muscle. So after reviewing the titles, abstracts and full texts, we excluded it.

IV. (PEDro score 8) Kamonseki DH, Gonçalves GA, Liu CY, Júnior IL. Effect of stretching with and without muscle strengthening exercises for the foot and hip in patients with plantar fasciitis: a randomized controlled single-blind clinical trial. Manual therapy. 2016;23:76-82. (balance)

A: Thank you for your suggestion. We only include research specific to intrinsic foot muscles training. The foot exercise group in this article utilizing extrinsic and intrinsic foot muscles. Given that the extrinsic foot muscles strengthening exercises may bring potential effect on the foot biomechanics outcome, so after reviewing the titles and abstracts, we excluded it.

V. (PEDro score 6) Lynn SK, Padilla RA, Tsang KK. Differences in static-and dynamic-balance task performance after 4 weeks of intrinsic-foot-muscle training: the short-foot exercise versus the towel-curl exercise. Journal of sport rehabilitation. 2012;21(4):327-33.

A: Thank you for your suggestion. The main aim of this study was to compare short-foot exercise and towel-curl exercise on foot function or dynamic postural balance, rather than explore the effect of IFMs training foot function and dynamic postural balance. So after reviewing the titles, abstracts and full texts, we excluded it.

3. There are two studies included that have a wrong year of publication. Please, correct it:

• Unver B, Erdem EU, Akbas E. Effects of Short-Foot Exercises on Fo 506 ot Posture, Pain, Disability and Plantar Pressure in Pes Planus. Journal of sport rehabilitation. 2019:1-16.

• Fraser JJ, Hertel J. Effects of a 4-week intrinsic foot muscle exercise program on motor function: a preliminary randomized control trial. Journal of sport rehabilitation. 2019;28(4):339-49.

A: Thank you for your suggestion. We have corrected it.

4. In the PROSPERO registry, the risk of bias assessment is described as PEDro scale. However, in this manuscript the authors did no use this scale but the Modified Downs and Black Quality Index. Please resolve this inconsistency with your PROSPERO registry and specify why the PROSPERO protocol was not adhered to. The reviewer #1 had already pointed it out in the first review but it seems the authors did not understand it and have not explained this issue.

A: Thank you for your suggestion. Even though we plan to utilize PEDro scale to assess the risk of bias, we finally decide to use Modified Downs and Black Quality Index to assess the methodological quality of the included studies based on the relative references, and we have added it to support our manuscript.

Ref:

1. *Huffer D, Hing W, Newton R, Clair M. Strength training for plantar fasciitis and the intrinsic foot musculature: A systematic review. Phys Ther Sport, 2017; 24:44-52.*
2. *Downs SH, Black N. The feasibility of creating a checklist for the assessment of the methodological quality both of randomised and non-randomised studies of health care interventions. J Epidemiol Commun H, 1998.*

5. Ref 35 is listed as “Kim EK, Kim JS. 2016” As authors but in the evidence table, it is listed as “Eun-Kyung et al. (2016)” Please correct that.

A: Thank you for your suggestion. We have corrected it.

6. Introduction Lines 53-54: ““Although numerous studies explore the effect of IFMs training, there is still lacking of evidence-based studies to review it systematically.” This sentence does not make sense. If there are many evidences, and it does indeed (you mentioned now in your paper) that proved the efficacy of the foot muscles training for several conditions, what exactly do you need? There is no “lacking evidenced-based studies”. On the contrary. What you really want is to review systematically the exiting evidences. Please, rephrase, again, the sentence.

A: Thank you for your suggestion. We have rephrased this sentence.

7. Introduction Lines 66-67: “Randomized controlled trials, intervention studies, were eligible for inclusion.” What are intervention studies? Non-controlled studies? Case studies? Interrupted case series? Please, use the SIGN (Scottish Intercollegiate Guidelines Network) methodology to describe the type of studies that were searched and included in your review.

A: Thank you for your suggestion. We have modified the description the type of studies according to the SIGN (Scottish Intercollegiate Guidelines Network) methodology, and added the relative reference to support our review.

Ref:

*Scottish Intercollegiate Guidelines Network (SIGN) Methodology Review Group, editor. Report on the review of the method of grading guideline recommendations. Edinburgh: SIGN 1999.*
