# Supplementary material for: Effect of intrinsic foot muscles training on foot function and dynamic postural balance: A systematic review and meta-analysis
Source: PLoS One. 2022 Apr 20;17(4):e0266525. doi: 10.1371/journal.pone.0266525 (PMC9020712; doi:10.1371/journal.pone.0266525)
Supplement: Attachment — Submitted filename: Response to Reviewers.docx [file pone.0266525.s006.docx]

**Dear Editor**

On behalf of our research team, we would like to thank the editor for giving us a chance to resubmit the manuscript (PONE-D-21-23366), and also thank the reviewers for giving us constructive suggestions, which would help us both in English and in depth to improve the quality of our study. Here we submit a new version of our manuscript with the title “Revised Manuscript with Track Changes”, which has been modified according to the reviewers’ suggestions. Revisions were highlighted using **red font** in manuscript. We also included our responses to the various points raised by the reviewer below.

We are looking forward to your reply.

Thank you.

Yours sincerely,

Lin Wang, MB, PhD

Professor,

Shanghai University of Sport

Shanghai, China.

Tel: (86) 21 5125 3426 Fax: (86) 21 5125 3470

Email: wanglin@sus.edu.cn

**Comments of Reviewer 2:**

The authors have written an interesting review paper that, through the peer-review process so far has made some obvious progress. Still, some major issues remain, as well as numerous minor issues. Major issues lie in the reproducibility and complexity of the methods and results. Still significant steps are required in these sections before they would be fit for publication. Further, there needs to be a clear match between the results collected here and the conclusions of the study, as the discussion section currently reads like a narrative literature review, without adding much analyses to the reviewed studies (the analyses seem isolated in the results and are not clearly linked to the discussion). I appreciate the further specification of the authors decision for using the Modified Downs and Black quality assessment to the manuscript. However, I do not feel the authors have sufficiently explained why they have neglected an a-priori established part of their protocol and chosen to use a different method. This undermines the credibility of the manuscript as one of the major strengths of a systematic review lies in the protocol that is established a-priori. As such, I would again ask the authors to explain in their response to reviewers letter why they made this decision.

A: Thank you for your efficient work in reviewing our manuscript. Your comments were highly insightful and help us improve the quality of the manuscript. We have modified our manuscript, and would like to re-submit it for your consideration.

Further comments in order of appearance.

Line 13-15. This sentence does not flow well due to the double use of the word ‘relevant’. I would suggest the following sentence: ‘Keywords related to IFM training were used to search four databases for relevant studies published between January 2011 and February 2021.’

A: Thank you for your suggestion. We have revised our manuscript follow your suggestion.

Line 34-36. This sentence does not flow well as it is unclear how ‘they’ should be interpreted. I would suggest rephrasing to something starting with: ‘The main IFMs are … ‘

A: Thank you for your suggestion. We have rephrased our manuscript and start with: ‘The main IFMs are …’

Line 45. So far this paragraph has been about running, but this final sentence switches the focus to walking. This breaks up the flow in the introduction.

A: Thank you for your suggestion. We have revised it.

Line 49-52. There is a flaw in the logic here. It cannot be stated that the part up until the first reference ‘[24]’ is proof for the part until the second reference ‘[23]’. These findings might be related, but proof is not offered by this statement.

A: Thank you for your suggestion. We have revised it.

Line 55-56. The aims statement does not flow logically from the previous. First, you state that much about the positive effects of IFM is already known. This is not a reason to study the concept further. A better rationale could be something along the lines of the following: ‘while a number of isolated studies have shown benefits of IFM, the applicability of these findings is still limited because, to date, no previous study has systematically studied these effects nor has a meta-analysis been applied to get an overall estimate of the effect of IFM training. Therefore, the current study aims…’

A: Thank you for your suggestion. We have revised our manuscript follow your suggestion.

Line 72. Consider changing ‘in accordance with’ to ‘based on’

A: We have corrected it.

Line 74-75. Please specify here what ‘desired foot biomechanical parameters’ were

A: Thank you for your suggestion. We have specified it.

Line 80. Consider ‘i.e.’ to ‘e.g.’

A: We have corrected it.

Line 87-91. This statement is confusing and needs to be rephrased.

A: Thank you for your suggestion. We have rewritten it.

Line 92-97. It is clear that the mean difference was taken as an outcome measure. However, it is unclear what this difference is based on, related to the different designs included in the study. For instance with an RCT. Was the difference of the experimental group between pre and post test used, or the difference between experimental and control group at post test?

A: Thank you for your suggestion. The mean difference is based on the difference between pre and post test used.

Line 170. Please report exact p values and associated tests statistics.

A: Thank you for your suggestion. We have reported exact p values.

Line 170-172. This sentence reads like a concluding sentence and therefore does not fit the results section.

A: We have deleted it.

Line 178-180. The authors conclude here that the funnel plot shows a ‘slight asymmetry’. What was the basis for this classification? That is, as a rule of thumb, 10 observations are required to statistically assess the asymmetry of a funnel plot. Unless the authors are aware of other guidelines, I think caution is warranted in this interpretation step. Especially considering that if one would scale figure 4B to have a X axis from -6 to 6, the asymmetry would appear way more drastic.

A: Thank you for your suggestion. We have modified our manuscript following your suggestion.

Line 184. The Eun-Kyung (2016) reference is not represented in table 1 and 2.

A: Thank you for your suggestion. Eun-Kyung (2016) has been revised as Kim et al [37] in table 1-3.

Line 184-187. This figure needs a clearer caption. What do the values indicate? Especially the ‘total’ column is not clear. I initially interpreted this as the N of the studies, but if this is true, then there are inconsistencies with table 2.

A: Thank you for your suggestion. We have modified the caption of figure, the ‘total’ column is the N of the studies, this typo has been corrected.

Table 3. Please specify the meaning of ‘NR’

A: We have added it.

Line 225. Which hypothesis is meant here? Discussion in general: this section reach much like a narrative review and the link to the quantitative results of this study should be emphasized.

A: Thank you for your suggestion. We have modified this section.

Line 279. Consider using ‘supervisor’ rather than ‘supervisors’

A: We have corrected it.
